# IncGraph: Incremental graphlet counting for topology optimisation

**DOI:** 10.1371/journal.pone.0195997

**Published:** 2018-04-26

**Authors:** Robrecht Cannoodt, Joeri Ruyssinck, Jan Ramon, Katleen De Preter, Yvan Saeys

**Affiliations:** 1 Data Mining and Modelling for Biomedicine group, VIB Center for Inflammation Research, Ghent, Belgium; 2 Center for Medical Genetics, Ghent University Hospital, Ghent, Belgium; 3 Cancer Research Institute Ghent (CRIG), Ghent, Belgium; 4 IDLab, Department of Information Technology, Ghent University – imec, Ghent, Belgium; 5 Department of Computer Science, KU Leuven, Belgium; 6 Department of Applied Mathematics and Computer Science, Ghent University, Ghent, Belgium; Janssen Research and Development, UNITED STATES

## Abstract

**Motivation:**

Graphlets are small network patterns that can be counted in order to characterise the structure of a network (topology). As part of a topology optimisation process, one could use graphlet counts to iteratively modify a network and keep track of the graphlet counts, in order to achieve certain topological properties. Up until now, however, graphlets were not suited as a metric for performing topology optimisation; when millions of minor changes are made to the network structure it becomes computationally intractable to recalculate all the graphlet counts for each of the edge modifications.

**Results:**

IncGraph is a method for calculating the differences in graphlet counts with respect to the network in its previous state, which is much more efficient than calculating the graphlet occurrences from scratch at every edge modification made. In comparison to static counting approaches, our findings show IncGraph reduces the execution time by several orders of magnitude. The usefulness of this approach was demonstrated by developing a graphlet-based metric to optimise gene regulatory networks. IncGraph is able to quickly quantify the topological impact of small changes to a network, which opens novel research opportunities to study changes in topologies in evolving or online networks, or develop graphlet-based criteria for topology optimisation.

**Availability:**

IncGraph is freely available as an open-source R package on CRAN (incgraph). The development version is also available on GitHub (rcannood/incgraph).

## Introduction

Even the simplest of living organisms already consist of complex biochemical networks which must be able to respond to a variety of stressful conditions in order to survive. An organism can be characterised using numerous interaction networks, including gene regulation, metabolic, signalling, and protein-protein interaction. The advent of high-throughput profiling methods (e.g. microarrays and RNA sequencing) have allowed to observe the molecular contents of a cell, which in turn have enabled computational network inference methods to reverse engineer the biochemical interaction networks [1]. Improving the accuracy of inferred networks has been a long-standing challenge, but the development of ever more sophisticated algorithms and community-wide benchmarking studies have resulted in significant process [2–5].

Several recent developments involve incorporating topological priors, either to guide the inference process [6] or post-process the network [7]. A common prior is that biological networks are highly modular [8], as they consist of multiple collections of functionally or physically linked molecules [9, 10]. At the lowest level, each module is made up out of biochemical interactions arranged in small topological patterns, which act as fundamental building blocks [11].

Graphlets [12] are one of the tools which could be used to add a topological prior to a biological network, Graphlets are small connected subnetworks which can be counted to identify which low-level topological patterns are present in a network. By comparing how topologically similar a predicted network is to what would be expected of a true biological network, a predicted network can be optimised in order to obtain a better topology.

**Fig 1:**
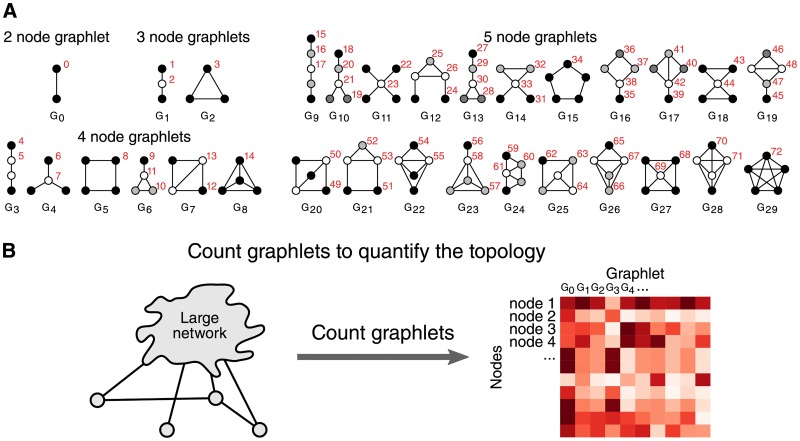
Graphlet counting in a network characterises its local topologies. (A) In total, there are 30 different graphlets containing 2 to 5 nodes, ranging from *G*_0_ to *G*_29_. Orbits are an extension of graphlets which also take into account the position of a node within a graphlet. The 73 different orbits are coloured in red. (B) By counting the occurrences of these graphlets in the network, the local topology surrounding a node can be quantified.

By counting the number of occurrences of each of the different graphlets (Fig 1A) touching a specific node, one can characterise the topology surrounding it. The graphlet counts of a network can be represented as a matrix with one row for each of the nodes and one column for each of the graphets (Fig 1B). An orbit represents a class of isomorphic (i.e. resulting in the same structure) positions of nodes within a graphlet (Fig 1A, coloured in red). Both graphlets and orbits have been used extensively for predicting the properties of nodes such as protein functionality [13–15] and gene oncogenicity [16], by performing network alignment [17, 18] or using them as a similarity measure in machine learning tasks [19, 20].

In this work, we focus on optimising gene regulatory networks by incorporating a topological prior as part of a topology optimisation process. We seek to optimise a predicted network by iteratively modifying the network and accepting modifications that lead to topological properties that resemble better those of true biological networks.

However, using graphlets to perform topology optimisation was hitherto not possible. Calculating the graphlet counts using the most state-of-the-art graphlet counting of a moderately sized gene regulatory network already has an execution time of about five seconds (*E*. *coli*, ∼ 3000 genes, ∼ 10000 interactions, up to graphlets up to 5 nodes). While this computational time poses no issue for regular static networks, recalculating all graphlet counts for every network modification made as part of a topology optimisation is computationally intractable. For example, performing 100’000 iterations of topology optimisation on a similarly sized network and calculating the topological impact of 10 possible edge modification at each iteration, already results in a computational time of about 12 days. Graphlet-based topology optimisation thus quickly becomes infeasible for larger networks.

When adding or removing an edge to a large network, the number of altered graphlets is much smaller than the total number of graphlets in the network. It could therefore be much more efficient to iterate over and count all the graphlets that have been added or removed as a result of the edge modification, than it is to recalculate the graphlet counts from scratch.

Eppstein et al. introduced data structures and algorithms for updating the counts of size-three [21] and size-four [22] subgraphs in a dynamic setting. The data structures were determined such that the amortised time is *O*(*h*) and *O*(*h*^2^), respectively, where *h* is the h-index of the network [23].

We developed IncGraph, an alternative algorithm and implementation for performing incremental counting of graphlets up to size five. We show empirically that IncGraph is several orders of magnitude faster at calculating the differences in graphlet counts in comparison to non-incremental counting approaches. In addition, we demonstrate the practical applicability by developing a graphlet-based optimisation criterion for biological networks.

## Materials and methods

Assume a network *G* of which the graphlet counts *C*_*G*_ are known. *C*_*G*_ is an *n*-by-*m* matrix, with *n* the number of vertices in the network, *m* = 73 is the number of different orbits, and where *C*_*G*_[*i*, *j*] is the number of times node *i* is part of a graphlet at orbit *O*_*j*_. Further assume that one edge has either been added or removed from *G*, resulting in *G*′, at which point the counts *C*_*G*′_ need to be observed. If multiple edges have been modified, the method described below can be repeated for each edge individually.

### Incremental graphlet counting

As stated earlier, recalculating the graphlet counts for each modification made to the network quickly becomes computationally intractable for larger network sizes. However, as the differences in topology between *G* and *G*′ are small, it is instead possible to calculate the differences in graphlet counts Δ_*G*, *G*′_ instead. This is much more efficient to calculate, as only the neighbourhood of the modified edges needs to be explored. *C*_*G*′_ can thus be calculated as *C*_*G*′_ = *C*_*G*_ + Δ_*G*, *G*′_.

**Fig 2:**
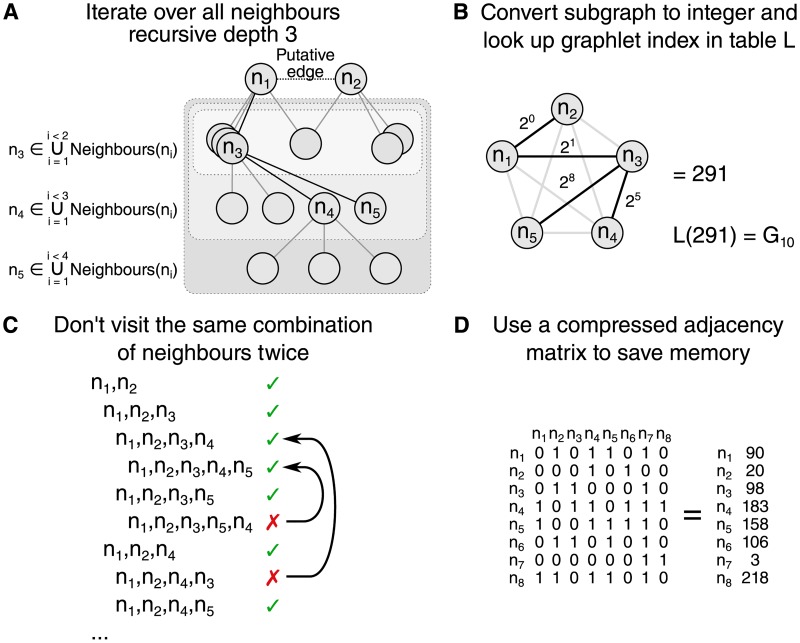
Several strategies are employed in order to reduce time and memory consumption. (A) Only the depth 3 neighbourhood of each modified edge needs to be explored in order to have visited all modified five-node graphlets. (B) A lookup table can be used to easily look up the graphlet index of a subgraph, by weighing each edge in a 5-node subgraph by a power of 2. (C) The same combination of five nodes is never repeated, as to avoid counting the same graphlet multiple times. (D) The adjacency matrix of the network is compressed in order to reduce memory usage.

The differences in graphlet counts Δ_*G*, *G*′_ are calculated by iterating recursively over the neighbours surrounding each of the modified edges (See S1 Pseudocode). Several strategies are used in order to calculate Δ_*G*, *G*′_ as efficiently as possible (Fig 2). (A) The delta matrix is calculated separately for each modified edge. Since the edge already contains two out of five nodes and any modified graphlet is a connected subgraph, the neighbourhood of this edge only needs to be explored up to depth 3 in order to iterate over all modified graphlets. (B) We propose a lookup table to look up the graphlet index of each node of a given subgraph. By weighting each possible edge in a 5-node graphlet, the sum of the edges of a subgraph can be used to easily look up the corresponding graphlet index. (C) During the recursive iteration of the neighbourhood, the same combination of nodes is never visited twice. (D) As the network can be relatively large, the adjacency matrix is binary compressed in order to save memory. One integer requires 4 bytes and contains the adjacency boolean values of 32 edges, whereas otherwise 32 booleans would require 32 bytes. For example, 1GB of memory is large enough to store a compressed adjacency matrix of 92681 nodes. If the network contains too many nodes to fit a compressed adjacency matrix into the memory, a list of sets containing each node’s neighbours is used instead.

IncGraph supports counting graphlets and orbits of subgraphs up to five nodes in undirected networks. By modifying the lookup table, the method can be easily extended to directed graphlets or larger-node graphlets, or limited to only a selection of graphlets. This allows for variations of the typical graphlets to be studied in an incremental setting.

### Timing experiments

We compared the execution time needed to calculate the graphlet counts in iteratively modified networks between our method and a state-of-the-art non-incremental algorithm, Orca [24]. Orca is a heavily optimised algorithm for counting 5-node graphlets in static networks, and outperforms all other static graphlet counting algorithms by an order of magnitude [24].

**Fig 3:**
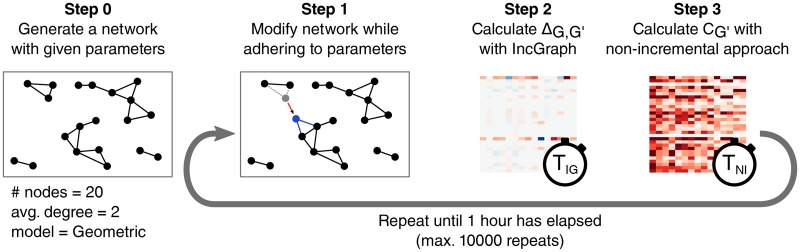
Static network model generators were modified to generate dynamic networks. Three network models were used: Barabási-Albert, Erdős-Rényi, and Geometric. Step 0: a network is generated according to the network model and the given parameters. Step 1: the network is modified such that the result is as likely to have been generated by the network model. Step 2: The time for IncGraph to calculate the differences in graphlet counts is measured (*T*_*IG*_). Step 3: The time for the non-incremental approach to calculate the graphlet counts of the new network is measured (*T*_*NI*_). Steps 1 to 3 are repeated until all modifications generated at step 0 are processed, or until an execution time threshold has been reached.

The timing experiments were performed by generating networks from 3 different network models, making modifications to those networks while still adhering to the network model, and measuring the execution times taken for both approaches to calculate the new graphlet counts (Fig 3).

The network models were based on three static network models: Barabási-Albert [25], Erdős-Rényi [26], and Geometric [27]. Pseudo code for these random evolving network models can be found in S2 Pseudocode, S3 Pseudocode, and S4 Pseudocode respectively. Each model generates an initial network according to the static network model it is based on, and a list of network modifications (removing an edge from or adding an edge to the network). These network modifications are made such that at any given time point in the evolving network, it is likely that the network at its current state could have been generated by the original static network model.

Networks were generated with varying network models, between 1000 and 16000 nodes, node degrees between 2 and 64, and 10000 time points. We measured the time needed to calculate the delta matrix at random time points until 1 hour has passed. All timings experiments were carried out on Intel(R) Xeon(R) CPU E5-2665 @ 2.40GHz processors, with one thread per processor. The generation of networks with higher node counts or degrees was constrained by the execution time of the network generators, not by IncGraph. All data and scripts are made available at github.com/rcannood/incgraph-scripts.

### Gene regulatory network optimisation experiments

We demonstrate the usefulness of IncGraph by using a simple graphlet-based metric to optimise gene regulatory networks. One of the striking differences between real and predicted gene regulatory networks is that the predicted networks contain highly connected subnetworks, which contain high amounts of false positives. We determine a penalty score such that predicted networks containing graphlets with many redundant edges will be penalised in comparison to very sparse networks.

**Fig 4:**
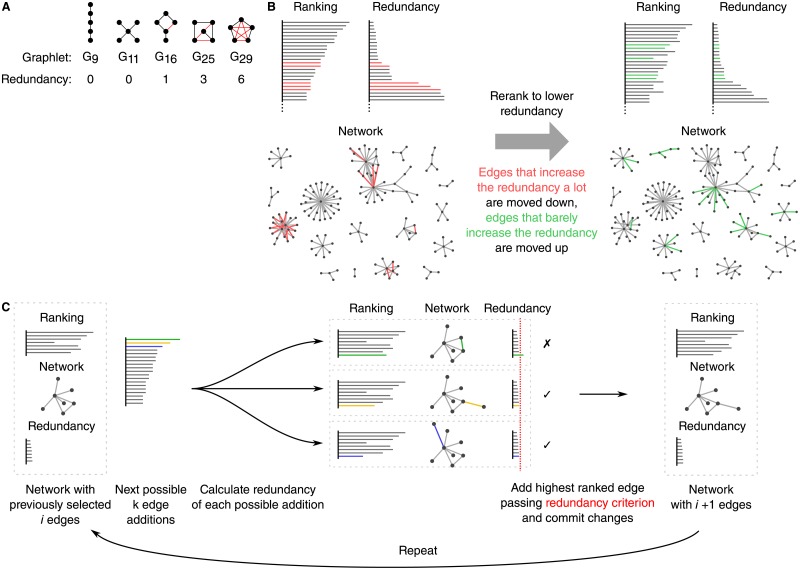
Predicted gene regulatory networks of model organisms are optimised to reduce the false positive rate. A) The number of redundant edges in each graphlet are counted. B) The network is optimised in order to obtain a lower redundancy over time. Two networks are shown, one before and one after the optimisation procedure. Edges coloured in red have been removed from the network after optimisation, green edges have been added. C) Starting from an empty network, the interactions are modified by iteratively evaluating the increase in redundancy of the next *k* interactions, and adding the first edge for which its redundancy is less than the 90^th^ percentile redundancy.

The *redundancy penalty* (Fig 4A) of a network is defined as the sum of occurrences of each graphlet multiplied by the redundancy associated with each individual graphlet. The redundancy of a graphlet is the number of edges that can be removed without disconnecting the nodes from one another. By using the redundancy penalty score, we aim to improve the gene regulatory network (Fig 4B).

The topology optimisation procedure uses an empty network as initialisation and grows the network by selecting interactions iteratively. Each iteration, the top *k* = 100 highest ranked interactions that are not currently part of the network are evaluated, and the highest ranked interaction passing the redundancy criterion is selected (Fig 4C). This procedure is repeated until a predefined amount of time has passed. As the aim of this experiment is not to obtain the highest performing topology optimisation method, parameter optimisation of *k* has not been performed and is considered to be outside the scope of this work.

We optimised gene regulatory networks of *E*. *coli* and *S*. *cerevisiae*. The predicted networks were generated using the network inference method GENIE3 [28] with default parameters. Gene expression data was obtained from COLOMBOS [29] and GEO [30], respectively. The predicted networks and the optimised versions thereof were compared against respective lists of known gene regulatory interactions [31, 32].

## Results and discussion

The contributions of this work are twofold. Firstly, we propose a new method for incrementally calculating the differences in graphlet counts in changing graphs, and show that it is orders of magnitude faster than non-incremental approaches. Secondly, we demonstrate its applicability by optimising a predicted gene regulatory network in order to reduce the false positive rate therein.

### Execution time is reduced by orders of magnitude

**Fig 5:**
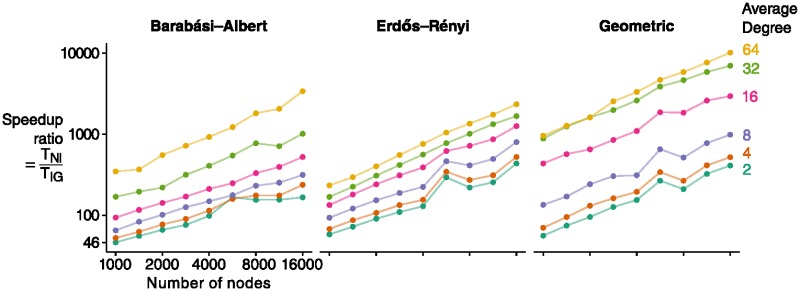
IncGraph is significantly faster than non-incremental approaches. For small networks, the execution time of IncGraph *T*_*IG*_ is already 50 times less than that of non-incremental approaches *T*_*NI*_. This ratio increases even further for networks with higher numbers of nodes or higher average degrees.

Timing experiments show that IncGraph is significantly faster in calculating the delta matrix in comparison to calculating the graphlet counts from scratch at each iteration (Fig 5). The observed speedup ratios between IncGraph and the non-incremental approach Orca ranges from about 50× to 10000×. The speedup ratio increases as the network size increases. For larger networks, IncGraph can thus calculate the delta matrices of 10000 edge modifications while the non-incremental approach calculates one graphlet count matrix.

Surprisingly, IncGraph obtains higher execution times for networks with 5657 nodes than for networks with 8000 nodes. One possible explanation is that the size of the data structures containing those networks are particularly favourable in avoiding cache misses. Confirmation of this explanation, however, would require further investigation.

Comparing the execution time of IncGraph to the h-index of the networks indicates that the amortised time of IncGraph could be *O*(*h*^3^) (S1 Fig). This is in line with the amortised times *O*(*h*) and *O*(*h*^2^) of the algorithm described by Eppstein et al. [22] for counting three-size and four-size subgraphs respectively.

### IncGraph allows for better regulatory network optimisation

**Fig 6:**
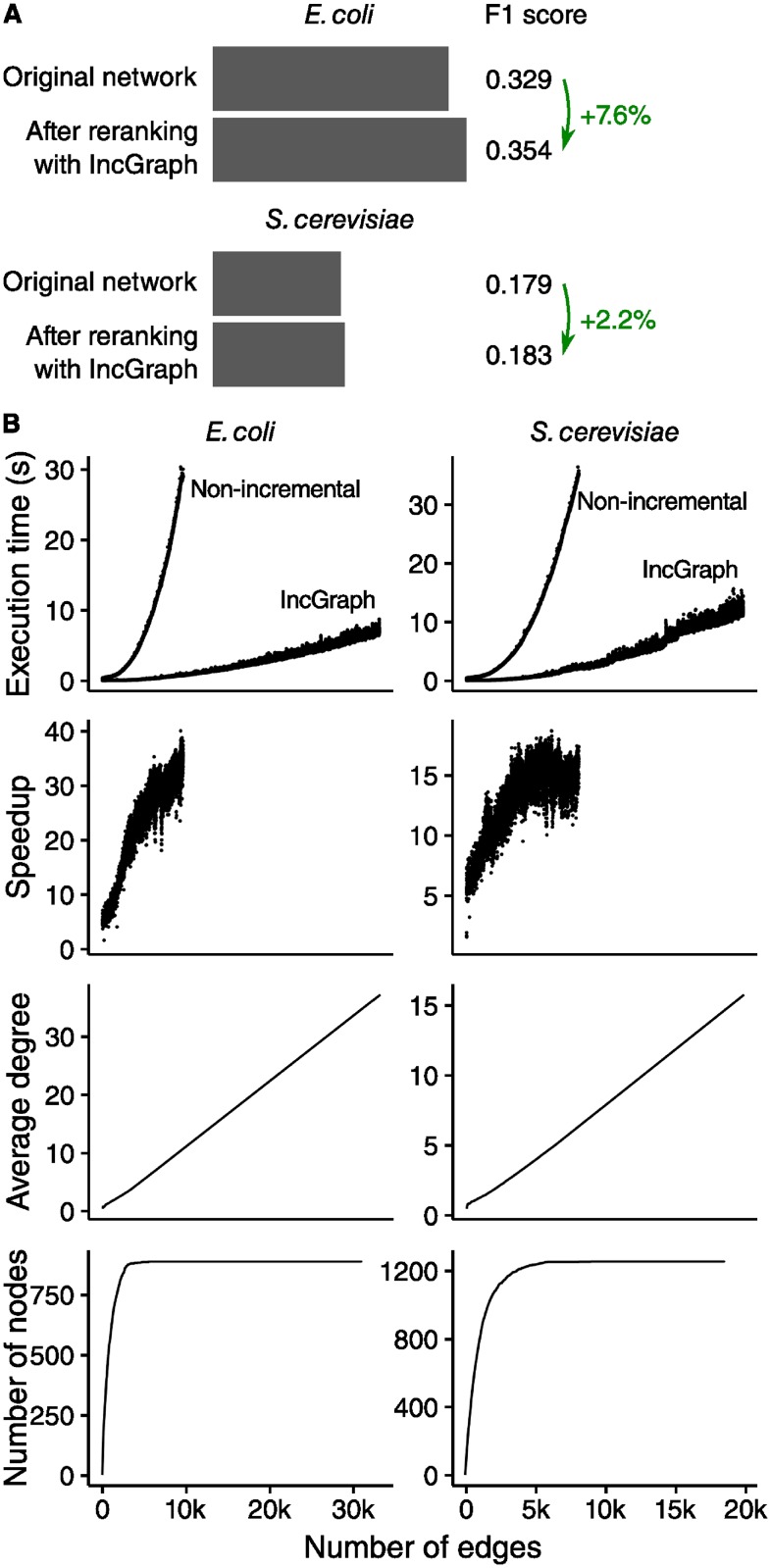
A simple graphlet-based scoring method improves predicted regulatory networks. (A) The F1 score was calculated by calculating the harmonic mean of the AUROC and AUPR scores of the first 1000 interactions. (B) IncGraph is significantly faster than the non-incremental approach. Note that for each interaction added to the network, the graphlet counts of 100 putative interactions were evaluated.

We implemented a graphlet-based optimisation algorithm for improving the false positive rate of the predicted gene regulatory networks of *E*. *coli* and *S*. *cerevisiae*. After reranking the regulatory interactions, the F1 score of the first 1000 interactions had increased by 7.6% and 2.2% respectively (Fig 6A). The obtained speedup of about 15–30× (Fig 6B) is in line with the experiments on *in silico* networks. Namely, for the *E*. *coli* network at 9618 interactions and 889 nodes (average degree = 10.8), a speedup of about 30× was obtained. Similarly, for the *S*. *cerevisiae* network at 8013 interactions and 1254 nodes (average degree = 6.4), a speedup of about 15× was obtained. These speedups are in the same order of magnitude of similarly sized networks (1000 nodes and 8000 interactions) generated with a Barabási-Albert model, with a speedup of 65×. This is to be expected, as such networks share the same scale-free property that gene regulatory networks have.

## Conclusion

Many improvements over the past few years have resulted in efficient graphlet counting algorithms, even for large static networks. However, needing to perform the simplest of tasks tens of thousands of times quickly becomes computationally intractable. As such, recalculating the graphlet counts of a network after each of the many network modification is infeasible.

This study introduces a method for calculating the differences in graphlet (and orbit) counts, which we call incremental graphlet counting or IncGraph for short. We show that IncGraph is at least 10–100 times faster than non-incremental methods for networks of moderate size, and that the speedup ratio increases even further for larger networks. To demonstrate the applicability of IncGraph, we optimised a predicted gene regulatory network by enumerating over the ranked edges and observing the graphlet counts of several candidate edges before deciding which edge to add to the network.

IncGraph enables graphlet-based metrics to characterize online networks, e.g. in order to track certain network patterns over time, as a similarity measure in a machine learning task, or as a criterion in a topology optimisation.

## Supporting information

S1 Pseudocode IncGraph calculates Δ_*G*, *G*′_ using a strict branch-and-bound strategy. (EPS)

S2 Pseudocode Pseudo code for generating an evolving Barabási-Albert (BA) network. It first generates a BA network, and then generates *o* operations such that at any time point, the network is or very closely resembles a BA network.
(EPS)

S3 Pseudocode Pseudo code for generating an evolving Erdős-Rényi (ER) network. It first generates an ER network, and then generates *o* operations such that at any time point, the network is or very closely resembles an ER network.
(EPS)

S4 Pseudocode Pseudo code for generating an evolving geometric network. It first generates a geometric network, and then generates *o* operations such that at any time point, the network is or very closely resembles a geometric network.
(EPS)

S1 Fig Empirical measurements show a strong relation between the execution time of IncGraph and the h-index cubed of the network it was applied to. This is in line with the findings by Eppstein et al., where counting 3-size subgraphs has an amortised time of *O*(*h*) and counting 4-size subgraphs has an amortised time of *O*(*h*^2^).
(EPS)
